# Promoting medical students’ reflection on competencies to advance a global health equities curriculum

**DOI:** 10.1186/1472-6920-14-91

**Published:** 2014-05-03

**Authors:** Patricia B Mullan, Joy Williams, Preeti N Malani, Michelle Riba, Andrew Haig, Julie Perry, Joseph C Kolars, Rajesh Mangrulkar, Brent Williams

**Affiliations:** 1 Department of Medical Education, University of Michigan Medical School, Ann Arbor, USA; 2 Department of Family Medicine, University of Michigan, Ann Arbor, USA; 3 Department of Internal Medicine, University of Michigan, Ann Arbor, USA; 4 Department of Psychiatry, University of Michigan, Ann Arbor, USA; 5 Department of Physical Medicine & Rehabilitation, University of Michigan, Ann Arbor, USA; 6 Department of Obstetrics and Gynecology, University of Michigan, Ann Arbor, USA

**Keywords:** Global health, Disparities, Medical education, Assessment, Reflection, Curriculum development

## Abstract

**Background:**

The move to frame medical education in terms of competencies – the extent to which trainees “can do” a professional responsibility - is congruent with calls for accountability in medical education. However, the focus on competencies might be a poor fit with curricula intended to prepare students for responsibilities not emphasized in traditional medical education. This study examines an innovative approach to the use of potential competency expectations related to advancing global health equity to promote students’ reflections and to inform curriculum development.

**Methods:**

In 2012, 32 medical students were admitted into a newly developed Global Health and Disparities (GHD) Path of Excellence. The GHD program takes the form of mentored co-curricular activities built around defined competencies related to professional development and leadership skills intended to ameliorate health disparities in medically underserved settings, both domestically and globally. Students reviewed the GHD competencies from two perspectives: a) their ability to perform the identified competencies that they perceived themselves as holding as they began the GHD program and b) the extent to which they perceived that their future career would require these responsibilities. For both sets of assessments the response scale ranged from “Strongly Disagree” to “Strongly Agree.” Wilcoxon’s paired T-tests compared individual students’ ordinal rating of their current level of ability to their perceived need for competence that they anticipated their careers would require. Statistical significance was set at p < .01.

**Results:**

Students’ ratings ranged from “strongly disagree” to “strongly agree” that they could perform the defined GHD-related competencies. However, on most competencies, at least 50 % of students indicated that the stated competencies were beyond their present ability level. For each competency, the results of Wilcoxon paired T-tests indicate – at statistically significant levels - that students perceive more need in their careers for GHD-program defined competencies than they currently possess.

**Conclusion:**

This study suggests congruence between student and program perceptions of the scope of practice required for GHD. Students report the need for enhanced skill levels in the careers they anticipate. This approach to formulating and reflecting on competencies will guide the program’s design of learning experiences aligned with students’ career goals.

## Background

In “Education of health professionals for the 21^st^ century, “ *Lancet* featured an international commission’s report that cited glaring gaps and inequities in health and health care outcomes, within and across nations, as constituting the most dramatic and incontrovertible evidence of the need to reform health education [1]. To achieve the health profession’s potential for ameliorating health disparities, the Commission recommended profound health education reform, moving it from the tradition of informative learning, focused on transmitting knowledge and skills to produce experts, and beyond formative learning, which focuses on socializing students around values to produce competent professionals. Instead, the Commission urges us to champion transformative learning, which focuses on developing leadership competencies intended to produce enlightened change agents capable of creatively adapting global resources to address priorities in local needs.

The proposed health professions education reform for ameliorating health disparities comes at a time when we can build on existing and emerging knowledge about conceptual frameworks for understanding global health [2] and health disparities [3], translational research practices bringing the potential for preventing or alleviating suffering into communities [4], educational practices fostering health professionals’ responsive leadership skills, [5] and aspirations of medical students for acquiring expertise in ameliorating health care disparities [6].

Among the stakeholders influencing the design and evaluation of new medical school programs, medical education accreditation agencies hold considerable sway. Prior to 1998, medical education accreditation agencies focused their expectations on instructional objectives as a way for medical schools and residency programs to make explicit to trainees and to the public the knowledge, behaviors, and attitudes that the programs intended learners to acquire. Associated assessment measures echoed this focus, parsing measures of knowledge, skills, and attitudes [7].

Lessons learned from reviews of research conducted on attitudes of medical students and residents suggest cautions for current efforts intended to promote accountability of health education in preparing trainees to ameliorate disparities. Rezler characterized medical education research as often framing attitudes of trainees as a problem that medical education might hope to redress [8]. In this tradition of approaching the study of attitudes of professionals in training as problems, studies of medical student and resident attitudes often documented declines in such valued professional orientations as altruism and empathy, with increases in cynicism, over the course of training. A recent review of longitudinal studies of empathy challenges the empirical evidence underlying the accepted wisdom about medical education’s insidious role in eroding attitudes assumed to inspire compassionate care, including empathic care of vulnerable populations [9]. In this review, Colliver’s re-analysis of the data showed that the extent of attitudinal changes to be slight and variable in direction. Colliver also challenged the construct validity of the measures, which often used diffuse statements about attitudes.

Medical schools have been urged to develop curricula in ways that are congruent with theories of motivating medical trainees’ learning [10]. Notable among these theories is Self-Determination theory, which emphasizes adults’ needs for competency, autonomy, and relationships [11]. A concurrent critique of existing research on self-assessment recommends that medical education adopt approaches more aligned with promoting learners’ reflection and self-directed assessment seeking [12].

Although accreditation agencies have moved to an emphasis on competencies to promote accountability, medical educators caution that the very general characterizations of competencies left important work to be done for meaningful curriculum development and associated assessment, including grounding expectations for training in the context of real work responsibilities [13], and making explicit the likely scope of practice [14].

Particularly for competencies not represented in traditional medical education, the scope of practice might not be explicit and might not be consistently valued as definitive and necessary, especially for trainees with limited access to role models in emerging scopes of practice. For example, in the Canadian (CanMEDS) accreditation system, advocacy (defined as physicians responsibly using “their expertise and influence to advance the health and well-being of individual patients, communities, and populations)” constitutes an explicit expectation for every member in the profession [15]. In the U.S. Accreditation for Graduate Medical Education (ACGME), advocacy is not explicitly defined as a goal of training for which a training program would be held accountable. Responses to Earnest’s [16] call to make advocacy, defined as “action by a physician to promote those social, economic, educational, and political changes that ameliorate the suffering and threats to human health and well-being that he or she identifies through his or her professional work and expertise” evoked markedly divergent responses, including protests that promoting advocacy would distort medical education [17].

Despite this ambivalence, medical schools are increasingly moving to provide instruction relevant to ameliorating global and domestic health disparities. A recent review of structured global health education programs in U.S. medical schools concluded that there was little standardization across programs in terms of their requirements for didactic, clinical, scholarly, and cultural components [18]. In this emerging field, the development of a self-assessment measure for capturing students’ skills and career aspirations for their roles could inform our collective ability to develop and evaluate responsive curricula.

In this study, we explore the insights drawn from a competency-based approach to assessing medical students’ perceptions of their level of competency related to practice in ameliorating disparities in domestic and global health settings. We elicit the level of competency students see themselves as bringing to the table as they initiate participation in a co-curricular medical school pathway, and compare this to the level of competency they perceive that their future careers will need. Our study questions were:

•what level of ability do medical students perceive themselves as having in defined competencies related to alleviating disparities, as they begin training for their careers?

•what level of ability do medical students perceive themselves as needing to have for their careers as physicians committed to alleviating disparities?

## Methods

In 2010, the Senior Associate Dean for Education at the university-affiliated study site charged the Assistant Dean for Medical Education with developing a co-curricular “pathway of customized, self-regulated learning” in Global Health and Disparities. Explicit in this charge were the expectations that the proposed program be aligned with the school’s social mission and extend existing learning and leadership opportunities for medical students related to ameliorating disparities, both nationally and globally. In addition to this explicit delegation of responsibility, the school’s administration also provided financial support equivalent to 10% full time equivalence to support the participation of a Director, six clinical faculty members, and a faculty member in medical education. The faculty members were experienced and actively involved in disparities research either in the U.S. or globally.

Members met weekly over 18 months. Working groups also included more than three dozen medical students interested in global health and disparities. The faculty planning group, working with students in an advisory role, reviewed existing research literature and publicly available descriptions of global health programs involving medical students. The planning group interviewed medical school faculty involved in applied global and domestic health care disparity work. Each faculty member was also encouraged to conduct informal key informant interviews with faculty from other colleges and universities that our medical students or planning faculty recommended for their leadership and active engagement in global health and disparities work. Each faculty member posted teaching resources for proposed competencies on a web-based site created for the project. A trained medical librarian with responsibility for supporting global health education and research at the study institution also conducted related searches of published resources, creating a dedicated information resource collection on our institution’s website. To facilitate the planning group’s curricular development efforts and planned student access to resources, the librarian also obtained institutional licenses for potential textbooks recommended by the planning faculty, posting electronic access to the textbooks the Program Director proposed. These include: *Medical management of vulnerable and underserved populations: Principles, practices, and populations*[19]; *Understanding global health*[20] and *Vulnerable Populations in the United States*[21].

We iteratively reviewed the set of competencies written or elicited by planning faculty members within the full planning group. Key sources for reviewing the proposed set of competencies also included the potential teaching resources posted on the group’s website. We piloted the statements of competencies for clarity and perceived importance with separate groups of first-year and fourth-year medical students working with our advisory group, with a subsequent review of the statements for clarity with a group of 12 clinical faculty members from diverse medical fields enrolled in our institution’s Medial Education Scholars Program We modified and synthesized statements of the competencies into a final set of 16 competency statements.

In 2012, 32 first-year medical students were admitted into a newly developed Global Health and Disparities (GHD) Path of Excellence. The GHD program is a set of mentored co-curricular activities built around defined competencies related to professional development and leadership skills to ameliorate health disparities in the United States and developing countries. To promote student reflection and inform the design of responsive educational curriculum, we asked our entering cohort to review the statements of competencies from two perspectives. First, GHD students rated their ability to perform the identified competencies that they perceived themselves as holding as they began their participation in the GHD program. Second, students identified the extent to which they considered their future career would require these responsibilities. For students’ assessments of both the level of capability they held as they entered the program and the extent to which their career would require these competencies, the response scale ranged from “Strongly Disagree” to “Strongly Agree.” Our institutional review board determined that this educational research qualified for human subjects exemption status. Our statistical analyses comparing students’ ordinal-level ratings of their current level of competence to the level they perceived they would need in their future career used Wilcoxin’s paired T-tests, with statistical significance set at p<. 01.

## Results

Tables 1 though 4 summarize the distribution of ratings from UM students accepted into the GHD program, in terms of the students’ self-assessed ability to now perform defined competencies related to GHD, compared to the level of competence they believe their career will require.

**Table 1:** Percent of students reporting current (“Now”) and needed (“Career Need”) competence in GHD-related competencies (n = 32)

		**% of students**				
**Domain: social determinants of health**		**Strongly disagree**	**Disagree**	**Agree**	**Strongly agree**	**Wilcoxin’s paired T-value**
		**1**	**2**	**3**	**4**	
Identify and apply frameworks describing the impact of society on the health of vulnerable populations, including differential distribution of diseases, risk factors, and health care access and delivery.	Now	6	34	56	3	4.5***
	Career Need	-	3	41	56	
Identify opportunities to affect societal determinants of health, drawing on approaches successfully used in ameliorating health disparities.	Now	6	38	56	-	4.4***
	Career Need	-	6	25	69	
Identify and employ strategies for resolving ethical issues arising in efforts to address health care disparity.	Now	-	50	41	9	3.9***
	Career Need	-	6	34	59	
Identify sources for funding research in health disparities, both domestically and internationally.	Now	28	47	19	6	4.7***
	Career Need	-	3	34	63	
Demonstrate cultural competence in learning and in developing and implementing programs related to health disparities.	Now	-	25	66	9	4.6***
	Career Need	-	-	25	75	

***P Value < .005.

### Ratings of current level of competencies within domains

Within the domain of Social Determinants of Health (Table 1), the competencies most students perceived themselves as being able to do related to demonstrating cultural competence (75% of students agreed/strongly agreed.) About half (59%) of the students characterized themselves as being able to identify and apply frameworks describing the impact of society on the health of vulnerable populations. In terms of being able to identify and use strategies to resolve ethical issues arising from efforts to address health care disparity, half of the GHD students thought they could do so; half indicated that could not. A majority (75%) felt that they could not presently identify sources for funding research in health disparities.

In contrast, fewer students rated their abilities related to health care systems and policy as responsibilities they could conduct (Table 2). Half of the students felt that they could identify and compare health systems in the U.S. and in a developing country, in terms of their use of organized and informal sectors; half could not. About half (47%) of the students felt that they could compare and contrast the role of non-governmental and governmental organizations in mitigating health disparities and improving health outcomes; 53% indicated they could not. Less than half of the students (43%) considered that they could compare and contrast health policies, priorities, and resources in different regions or nations. Only a third of students felt that they could use a range of health information systems to inform their work in populations with health disparities.

**Table 2:** Percent of students reporting current (“Now”) and needed (“Career Need”) competence in GHD-related competencies (n = 32)

		**% of students**				
**Domain: health care systems and policy**		**Strongly disagree**	**Disagree**	**Agree**	**Strongly agree**	**Wilcoxin’s paired T-value**
		**1**	**2**	**3**	**4**	
Compare and contrast health policies, priorities, and resources in different regions or nations.	Now	12	44	34	9	4.0***
	Career Need	-	9	28	62	
Compare and contrast the roles of non-governmental and governmental organizations in mitigating health disparities and improving health outcomes.	Now	6	47	41	6	4.4***
	Career Need	-	6	22	72	
Identify and compare health systems in the U.S. and in a developing country, in terms of their use of organized and informal sectors.	Now	9	41	41	9	3.3***
	Career Need	-	18	38	44	
Identify and demonstrate your ability to use a range of health information systems to inform your work in populations with health disparities.	Now	16	50	34	-	4.4***
	Career Need	-	6	32	61	

***P Value < .005.

Table 3 summarizes students’ ratings of their level of competence related to strategies to promote sustainable change. Defining sustainability was the only competency within this domain that elicited ratings from even a slight majority (62%) of students indicating that they could do it. Less than half (40%) of students felt that they understood health communication methods and technologies for improving health behavior and outcomes. Most (81%) of students characterized themselves as currently unable to critically review and analyze elements of successful capacity building in the context of a health disparities problem.

**Table 3:** Percent of students reporting current (“Now”) and needed (“Career Need”) competence in GHD-related competencies (n = 32)

		**% of students**				
**Domain: strategies to promote sustainable change**		**Strongly disagree**	**Disagree**	**Agree**	**Strongly agree**	**Wilcoxin’s paired T-value**
		**1**	**2**	**3**	**4**	
Describe and implement key steps in program development and evaluation, including needs assessment, identifying health markers and project resources, community and stakeholder engagement, monitoring program implementation, and identifying program impact.	Now	25	66	9	-	4.9***
	Career Need	-	9	34	56	
Identify effects of a specific internal-external health partnership on the target community as a whole, beyond the immediate intended health effects.	Now	12	72	16	-	4.8***
	Career Need	-	6	41	53	
Define sustainability, how it can be measured, and apply at least one element to a health disparities project to enhance its sustainability.	Now	3	34	56	6	3.2***
	Career Need	-	9	50	41	
Critically review and analyze elements of successful capacity building in the context of a health disparities problem.	Now	6	75	16	3	4.6***
	Career Need	-	6	68	26	
Demonstrate understanding of health communication methods and technologies for improving health behavior and outcomes.	Now	3	56	34	6	4.4***
	Career Need	-	3	34	62	

***P Value < .005.

In terms of students’ professional and leadership development (shown in Table 4), students were almost evenly split in characterizing their current ability levels. About half (51%) characterized themselves as currently understanding organizational leadership, including working with teams and aligning projects with institutional missions. Somewhat fewer students (41%) characterized themselves as currently able to formulate a plan for their personal ongoing involvement, career advancement, and training in the field of health disparities.

**Table 4:** Percent of students reporting current (“Now”) and needed (“Career Need”) competence in GHD-related competencies (n = 32)

		**% of students**				
**Domain: professional and leadership development**		**Strongly disagree**	**Disagree**	**Agree**	**Strongly agree**	**Wilcoxin’s paired T-value**
		**1**	**2**	**3**	**4**	
Demonstrate understanding of organizational leadership, including leadership styles and strategies, working with teams, and aligning projects with institutional missions.	Now	3	45	48	3	4.4***
	Career Need	-	-	38	62	
Present a plan for personal ongoing involvement, career advancement, and training in the field of health disparities.	Now	9	50	38	3	4.2***
	Career Need	3	3	28	66	

***P Value < .005.

### Ratings across domains of competence

Across domains, on 10 of the 16 competencies, student’s ratings ranged across the full spectrum of “strongly disagree” to “strongly agree” that they could perform the defined GHD-related competencies. However, on most (11) of the 16 competencies, at least 50% of students “disagreed” or “strongly disagreed” that they could perform the stated competence, which indicated that the stated competencies were beyond their present ability level.

Competency statements most often eliciting student ratings that they “disagreed” or “strongly disagreed” that they *now* could perform the proposed GHD-related responsibility included: describing and implementing key steps in program development and evaluation; identifying effects of internal-external health partnership on the target community as a whole, beyond the immediate intended health effects; identifying sources for funding research in health disparities, both domestically and internationally; critically reviewing and analyzing elements of successful capacity building in the context of a health disparities problem; identifying and demonstrating one’s ability to use health information sources to inform work in populations experiencing health disparities; presenting a plan for personal ongoing involvement, career advancement, and training in health disparities.

In contrast, at least 50% of GHD students reported that they “agreed” that they could now: identify and employ strategies for resolving ethical issues arising in efforts to address health care disparity; demonstrate understanding of organizational leadership, including leadership styles and strategies, working with teams, and aligning projects with institutional missions; demonstrate cultural competence in your learning and in developing and implementing programs related to health disparities; identify and compare health systems in the U.S. and in a developing country, in terms of their use of organized and informal sectors; define sustainability, how it can be measured, and apply at least one element to a health disparities project to enhance its sustainability; identify opportunities to affect societal determinants of health, drawing on approaches successfully used in ameliorating health disparities; and identify and apply frameworks describing the impact of society of the health of vulnerable populations, including differential distribution of diseases, risk factors, and health care access and delivery.

### Ratings of anticipated need for GHD-related competencies in their career

For 15 of the 16 defined competencies, 90% of the GHD student-participants “agreed” or “strongly agreed” that their careers would require them to be able to conduct the defined competency. The sole exception was the ability to identify and compare health systems in the U.S. and in a developing country, in terms of their use of organized and informal sectors, as 18% of students “disagreed” that their career would require this competency.

All (100%) of GHD students characterized their careers as requiring competence in both organizational leadership and in cultural competence as they work to develop and implement program related to health disparities. Only two students “disagreed” or “strongly disagreed” that their future careers would require them to formulate a plan for personal ongoing involvement, career advancement, and training in the field of health disparities. In contrast, 93.3% of GHD students “agreed” or “strongly agreed” their future careers would require them to develop such personal development and career planning.

### Comparison of current ability to perceived need for GHD-related competencies

Each table summarizes the results of statistical analyses (Wilcoxin’s paired T-test, the nonparametric equivalent to the paired t-test statistical analysis) comparing individual students’ rating of their current level of ability to their perceived need for competence that they anticipate their career will require.

For each of the 16 competencies, the results of Wilcoxin paired T-test values indicate that students perceive more need in their careers for the GHD-program defined competencies than they currently possess, at statistically significantly levels.

## Discussion and conclusion

In this study, we explored the use of explicit competencies to promote medical students’ reflection on the depth and scope of global health equity related expertise. While the results show that some students characterize themselves as already having at least some ability to conduct these competencies, the findings also indicate that students see the need for higher levels of skills in the careers they anticipate. The finding that the GHD founding student cohort rated the full set of potential competencies as germane to the careers they anticipate suggests considerable congruence between student and faculty perceptions of the scope of practice required for GHD.

Limitations of this study include that it reflects its use in a single study institution. The methods for identifying competencies drew on the resources of faculty involved in global and domestic health disparities research, who received institutionally supported time for their efforts in developing the curriculum and associated assessments. Although we sought to extend what we could build from drawing on their expertise with reviews of literature and informal key informant interviews with colleagues from other schools and professions, the collegial building of the network of faculty who would continue to work on the project would be less appropriate for global health curriculum development intended to minimize the influence of faculty perceived to have more expertise and authority, for which such methods as the Delphi approach to anonymously gathering and reviewing formal ratings of priorities would be appropriate. In addition, although the pilot-testing of the assessment instrument for clarity included medical school faculty not involved in global health, the findings obtained in this study draw on the instrument’s use with a special group of medical students interested in pursing global health and disparities training, so the findings might not be generalizable to other populations. We also recognize that students’ self-assessments of their competence in cultural competence can’t be considered evidentiary of their actual ability to practice, but recognizing their perception can inform the ways that we develop curriculum to test and enhance their skills. Further, the small number of students involved in our entry cohort of GHD students precludes the use of factor analysis, the statistical analysis that could be used to confirm the clustering/organization of competencies within such construct domains as leadership and social determinants of health. Despite the relatively small number of students involved, the magnitude of the effect size was sufficiently large that the comparison of current and needed competency was statistically significant.

Our program evaluation approach (formulating GHD-related practice competencies and eliciting students’ perceived current level of ability and the level of ability their future careers will require) provides several advantages. First, we believe that this practice promotes reflection - of both the faculty and students - about the scope of practice that could inspire student’s instruction. This is an important consideration, particularly in developing expertise required for careers that have not historically been a focus of medical training programs. Second, this approach provided empirical evidence that some GHD students already perceived themselves as having significant training and experience related to GHD competencies. This recognition influenced our curriculum’s development of mentored learning activities that students with existing commitment, training, and experience related to GHD competencies could lead. This informed curriculum design decisions to make most large group didactic sessions optional, give greater emphasis to case-based discussion groups for which students rotated leadership roles, and conduct and report on focused field work based on topics small groups of students selected.

The practice of eliciting students’ perceptions of their current level of skills can inform the program about skills students perceive that they already command.

Both curricular programs and their associated assessment systems can draw on the theoretical framework of Self-Determination Theory, for which strong empirical research support exists, across patient and professional health education applications [22]. Existing research has shown that training programs that provide medical students and residents with opportunities for informed advocacy can have important health amelioration outcomes [23,24].

Medical education programs promoting reflection of medical students drawn to these programs are consistent with theories of learning and motivation for medical professionals [10,22]. Such approaches can inform responsive curriculum development and career planning that sustains inspirations for advocacy in medical careers.

### Ethics committee

The University of Michigan’s Human Research Protection Program, which directs the Institutional Review Board, deemed the study exempt, assigning the study identification number HUM00070661.

## Competing interests

The authors have no competing interests to disclose.

## Authors’ contributions

Study’s conception and design: PBM, JW, MR, AH, JS, JK, RM, BW. Study’s acquisition of data: PBM, JW, PNM, BW, JS. Analysis of data: PBM, PNM, BW. All authors involvement in drafting the manuscript and revising it for critical intellectual content and approval of the manuscript.

## Pre-publication history

The pre-publication history for this paper can be accessed here:

http://www.biomedcentral.com/1472-6920/14/91/prepub
